# Prediction of remaining useful life (RUL) of Komatsu excavator under reliability analysis in the Weibull-frailty model

**DOI:** 10.1371/journal.pone.0236128

**Published:** 2020-07-15

**Authors:** Awat Ghomghaleh, Reza Khaloukakaie, Mohammad Ataei, Abbas Barabadi, Ali Nouri Qarahasanlou, Omeid Rahmani, Amin Beiranvand Pour

**Affiliations:** 1 School of Mining, Petroleum & Geophysics Engineering, Shahrood University of Technology, Shahrood, Iran; 2 Department of Engineering and Safety, UiT The Arctic University of Norway, Tromsø, Norway; 3 Department of Natural Resources Engineering and Management, School of Science and Engineering, University of Kurdistan Hewlêr (UKH), Erbil, Kurdistan Region, Iraq; 4 Institute of Oceanography and Environment (INOS), Universiti Malaysia Terengganu (UMT), Kuala Nerus, Terengganu, Malaysia; Univerza v Mariboru, SLOVENIA

## Abstract

It is an essential task to estimate the remaining useful life (RUL) of machinery in the mining sector aimed at ensuring the production and the customer’s satisfaction. In this study, a conceptual framework was used to determine the RUL under the reliability analysis in a frailty model. The proposed framework was implemented on a Komatsu PC-1250 excavator from the Sungun copper mine. Also, the Weibull-frailty model was selected to describe the failure behavior and compare it with the classical-exponential model. The frailty model was also used to evaluate the impact of unobserved environmental conditions on the RUL values. Both applied models were fitted to the obtained data from 80 operational hours of the Komatsu PC-1250 excavator. Plotting the results from the reliability analysis of two models demonstrated that the mine system with the frailty model performs better than the classical model before reaching the reliability of 80%. Besides, the frailty model shows a coherent with the operational time of the excavator, while the classical model demonstrates a sinusoid variation. The obtained results may be used for the development of maintenance, preventive repairs planning, and the spare parts replacement intervals.

## Introduction

The mining machinery sector of Iran has been faced with numerous difficulties in terms of the production capacity and cost to compete in global markets. Problems arise, however, when the opportunities are foreclosed in Iran as a worldwide attempt to implement the sanction policy [1]. The existing accounts fail to resolve these difficulties, and a focus on new indicators and approaches needs to be associated with higher efficiency in the mining machinery sector of Iran.

Indicators such as reliability, availability, and security are recognized as significant factors to determine the efficacy of the mining system. On the other hand, heavy-maintenance costs cause a complicated process in the mining system, especially mining engineers, who would expand a support system with prevailing conditions. There would, therefore, seem to be a definite need for changing the traditional approaches and preventing heavy-maintenance costs.

Failure prognosis, which is to predict the occurrence of impending failure before its occurrence, can be used as one of the novel approaches in the mining system. Failure prognosis approach estimates the occurrence risk in different states of failure in the future and predicts the remaining useful life (RUL) of the system. Indeed, the RUL estimates the occurrence time of the failure based on prevailing conditions in the system [2]. In other words, the past behavior of the mining system is signified by using the RUL, which is indicated by the reliability analysis. As a result, the failure prognosis approach provides an estimation of the RUL by detecting the signs of failure on the maintenance schedule [3, 4]. Recently, engineers have applied the RUL more widely in maintenance planning, checking system performance, spare parts estimation, and product promotion.

In the reliability analysis, the remaining lifetime of a component after the time ‘*t’* is considered as mean residual life (MRL). This function was first proposed by Watson and Wells [5] to analyze failures during the infancy period (Bathtub Curve). Elsayed [6] studied the MRL of industrial furnace pipes and proposed a method of estimating the MRL value based on the pipe's reliability level. This method has also sought to determine the optimal environmental conditions for pipes at the lowest expense [6]. Xie et al. [7] examined the change points of failure rate and the MRL function and resulted that the failure rate is decreased by increasing the MRL. Another study conducted by Xie et al. [8] shows that a flat portion occurs in the middle of the Bathtub curve equivalent to the distance between the change points. Along with the evaluation of the RUL function in different industries, however, a model is required to have a flat portion that is long in the mining system.

Liu et al. [9] proposed a reliability-oriented algorithm for predicting the RUL of rotary machine bearings. The temperature and vibration signals are used in this algorithm, followed by modeling the behavior of the vibrational time series by fuzzy logic. For the first time, Ghodrati et al. [10] applied the reliability-based RUL to analyze the residual life of hydraulic jacks in LHD loaders in the Swedish underground mines. Although a model proposed by Ghodrati et al. [10] coincides with environmental conditions, it suffers from an accuracy estimation of the RUL in terms of data availability. Therefore, this study seeks to obtain data for estimating the RUL in the Sungun copper mine, which will help to address this research gap. To this aim, a combination of Weibull analytical function [11] and fuzzy logic is employed, and the results from the combined model are used for the maintenance planning.

The present study has several practical applications. Firstly, the research results represent a further step towards developing the RUL determination in the mine system like Sungun copper mine, while previous studies are limited by weak designs and a failure to address the RUL determination in the field of mining engineering. Secondly, it points to the steps after determining the RUL, as far too little attention has been paid to the RUL in the mine system. The third and most crucial point is that the frailty model has not been studied in the mine system, especially in the RUL determination. Thus, this study provides a novel approach to integrating a frailty model in the RUL determination in the Sungun copper mine, Ahar, Iran.

## Methods

### Reliability analysis

Different types of mechanical failures were considered in the reliability analysis, such as instability, shear loading, crushing, and bending [12]. Failure was defined as the inability of an item to timely perform the expected activity [13]. In the reliability analysis, these data were collected in the form of time between failures (TBF), time to failure (TTF), the time between maintenance (TBM), and in the case of reparability topics in the form of time to repair (TTR), time to corrective maintenance (TCM), time to do preventive maintenance (TPM), and procurement and management stoppage times (TTDs). These data were also divided into two categories: completed failure data and suspended data [12].

**Fig 1:**
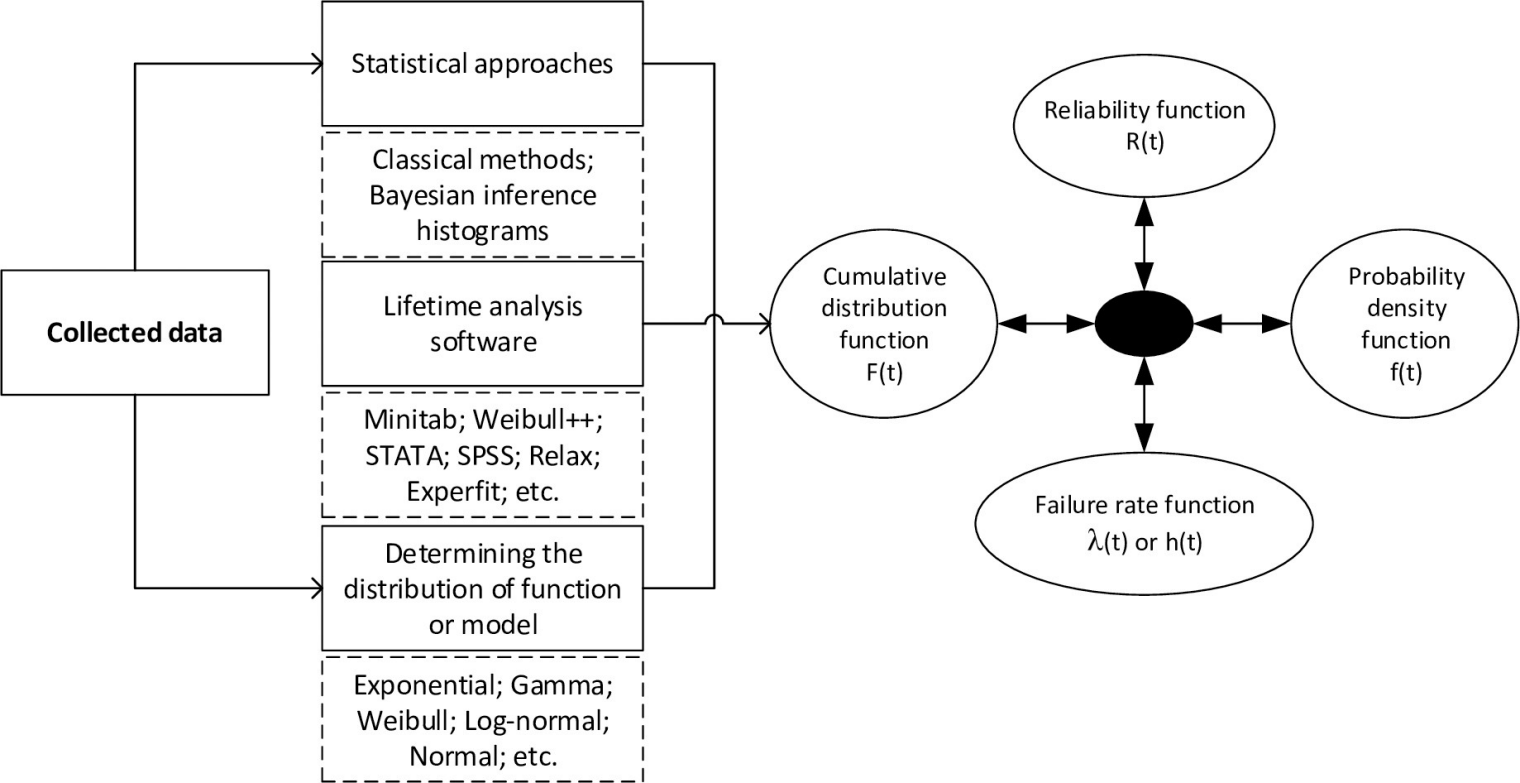
Data processing diagram in the reliability analysis; reprinted from Diallo et al. [14].

Four different functions were statistically defined to describe the failures as follows: (1) failure distribution known as probability density function (PDF) with the symbol f(t), (2) cumulative distribution function (CDF) with the symbol F(t), (3) the co-function of F(t) called the reliability function with the symbol R(t), and (4) the failure rate function or the hazard function with the symbol λ(t) or h(t). As shown in Fig 1, after entering the data, the relevant software was selected in the first step of the appropriate statistical approach [14]. Then, the proper function or model of one of the main functions (for example, f(t)) was fitted. Therefore, the next three functions F(t), R(t), and λ(t) can be calculated using the fitted function [15].

The hazard rate was considered to be the rate of occurrence of failures over a specified time (t_1_, t_2_). This rate was defined as the probability of occurring failure per unit time of the interval (t_1_, t_2_) so that the failure has not occurred before t_1_ (beginning of the interval) [16, 17]. The system reliability was defined as the ability of the mine system to perform and maintain the necessary functions under certain conditions without the occurrence of the failure during the specified time [18]. Eq (1) was applied to mathematically define the system reliability (R(x)) [15]:

$$R\left(x\right)=1-F\left(t\right)=1-\int_{0}^{t}f\left(x\right)\;dx$$ (1)

**Fig 2:**
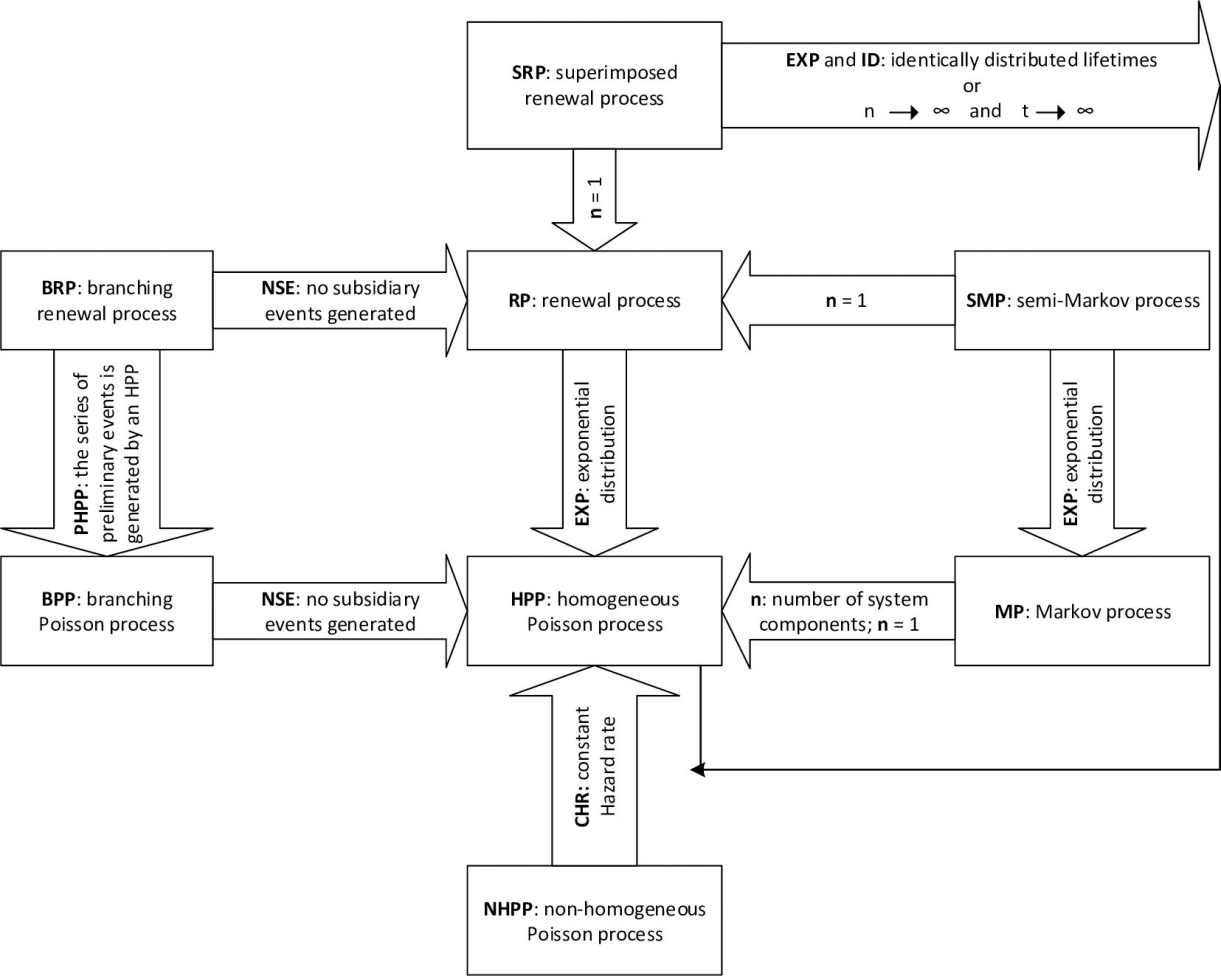
The relationship between classical statistical methods; reprinted from Pijnenburg [19].

Where R(x) denotes the system reliability (%) at the time *t*. Also, the processes used in the reliability analysis were included: homogeneous Poisson process (HPP), renewal process (RP), non-homogeneous Poisson process (NHPP), superimposed renewal process (SRP), branching renewal process (BRP), branching Poisson process (BPP), Markov process (MP), and semi-Markov process (SMP). The relationship between these processes was shown in Fig 2.

From Fig 2, *n* represents the number of the system components (in SRP) or the number of states (in MP and SMP). Besides, the mean time to the occurrence of failure [20] in the mine system is called the meantime (leading) to failure or MTTF (in short), which can be defined as Eq (2).

$$MTTF=\int_{0}^{\infty }\frac{\text{f}\left(\text{t}\right)}{1-\text{F}\left(\text{t}\right)}$$ (2)

The main difference between MTTF and MTBF was referred to their applications. The MTBF was used in the case of repairable systems instead of the MTTF. The MTBF was considered as the average time between a system’s failures while the MTTF was used in the case of unrepairable failure [15].

### Frailty model

The frailty model was referred to unobserved risk factors such as the impact of management on the progress of the mine system. However, ignoring observed risk factors such as rock type and climatic conditions causes unbiasedness in the results [21, 22]. Therefore, the evaluation of unobserved risk factors seems to be essential in the reliability analysis. To this aim, Eq (3) was applied to integrate unobserved risk factors in the frailty model [23–25].

λ(t|α)=αλ(t) (3)

From Eq (3), λ(t) is the rate of fundamental hazard that depends on time. *λ*(*t*|*α*) is the conditional hazard rate in the frailty model. α denotes the frailty as a positive random quantity, assumed to have a mean 1*θ* variance. For values of *α* >1, the frailty increases the risk of failure, and it would be vice versa for values of *α* < 1. The relationship between frailty and reliability can also be achieved based on the relationship between the conditional hazard rate and the reliability function (R(t)). The conditional reliability function (*R(t|α)*) depends on the frailty of (α) and can be expressed as Eq (4).

$$R\left(t|\alpha \right)=exp\left\{-\int_{0}^{t}\lambda \left(u|\alpha \right)du\right\}=exp\left\{-\alpha \int_{0}^{t}\frac{f\left(u\right)}{R\left(t\right)}du\right\}={\left\{R\left(t\right)\right\}}^{\alpha }$$ (4)

The non-conditional reliability function can also be estimated by integrating the value of (α) in the frailty model. Eq (5) shows an integrated function [26] between the PDF of (α) and the gamma (g(α)), which has a mean of 1^*θ*^ variance.

$$g\left(\alpha \right)=\frac{\alpha^{\frac{1}{\theta }-1}e^{-\frac{\alpha }{\theta }}}{Г\left(\frac{1}{\theta }\right)\theta^{\frac{1}{\theta }}}$$ (5)

In this case, the conditional reliability function [26] was obtained from Eq (6).

$$R_{\theta }\left(t\right)=\int_{0}^{\infty }{\left\{R\left(t\right)\right\}}^{\alpha }g\left(\alpha \right)d\alpha$$ (6)

In Eq (7), the subscript θ has been used for R to emphasize the dependence of frailty on the variance θ [26].

$$R_{\theta }\left(t\right)={\left[1-\theta ln\left\{R\left(t\right)\right\}\right]}^{\frac{-1}{\theta }}$$ (7)

### RUL estimation

The RUL was widely used in reliability-based research [27]. The RUL in the mine system was considered as the remaining time of proper operation before the occurrence of the failure. The estimation of the RUL was recognized as a critical factor for condition-based maintenance (CBM) [28–30]. According to Mazhar et al. [29], RUL estimation plays a crucial role in reusing and recycling raw materials and products. The lifespan of products or raw materials [31] must be high enough that they can be reused or recycled. This point highlights the RUL estimation for the CBM. The RUL was calculated using Eq (8). The mean residual life (L) is the average expected time to the occurrence of the failure in the mine system with a lifespan of *t*_0_ [10].

$$L\left(t\right)=E\left(T-t|T\geq t\right)=MTTF\left(t_{0}\right)=\int_{t_{0}}^{\infty }\left(\text{t}-t_{0}\right)\text{f}\left(\text{t}|t_{0}\right)\text{dt}\;t\geq 0$$ (8)

$$f\left(t|t_{0}\right)=h\left(t\right)\times R\left(t|t_{0}\right)\;\text{t}>t_{0}$$ (9)

From Eqs 8 and 9, *f(t|t*_*0*_*)* is the conditional function of the PDF at time *t*, provided that the failure has not occurred until the time *t*. *R(t|t*_*0*_*)* is the conditional reliability of the mine system until the time *t* that the failure does not happen in the mine system. In this case, the mine system was remained intact until the time *t*_*0*_. Thus, Eq (9) can be written as Eqs (10) and (11).

$$f\left(t|t_{0}\right)=h\left(t\right)\times \;\frac{R\left(t\right)}{R\left(t_{0}\right)}$$ (10)

$$f\left(t|t_{0}\right)=\frac{f\left(t\right)}{R\left(t_{0}\right)}\;\text{t}>t_{0}$$ (11)

Using Eqs (8) and (11), we would have:

$$MTTF\left(t_{0}\right)=\int_{t_{0}}^{\infty }t\times \frac{f\left(t\right)}{R\left(t_{0}\right)}dt-\int_{t_{0}}^{\infty }t_{0}\times \frac{f\left(t\right)}{R\left(t_{0}\right)}dt$$ (12)

For the second part of Eq (12), we have:

$$\int_{t_{0}}^{\infty }t_{0}\times \frac{f\left(t\right)}{R\left(t_{0}\right)}\text{dt}=\frac{t_{0}}{R\left(t_{0}\right)}\int_{t_{0}}^{\infty }\text{f}\left(\text{t}\right)\text{dt}=\frac{t_{0}}{R\left(t_{0}\right)}\left[F{\left(t\right)}_{t_{0}}^{\infty }\right]=\frac{t_{0}}{R\left(t_{0}\right)}\left[1-F\left(t_{0}\right)\right]=\frac{t_{0}}{R\left(t_{0}\right)}\left[R\left(t_{0}\right)\right]=t_{0}$$ (13)

Using Eqs (12) and (13), we will have:

$$MTTF\left(t_{0}\right)=\frac{1}{R\left(t_{0}\right)}\int_{t_{0}}^{\infty }\text{t}\times \text{f}\left(\text{t}\right)\text{dt}-t_{0}$$ (14)

By expanding the integral of Eq (14), we would have:

$$MTTF\left(t_{0}\right)=\frac{1}{R\left(t_{0}\right)}\left(\int_{0}^{\infty }\text{t}\times \text{f}\left(\text{t}\right)\text{dt}-\int_{0}^{t_{0}}\text{t}\times \text{f}\left(\text{t}\right)\text{dt}\right)-t_{0}$$ (15)

From Eq (15), the first integral is the same MTTF $\left(MTTF=\int_{0}^{\infty }t\times f(t)dt\right)$. For the second integral, if we rewrite f(t) based on R(t), we will have:

$$f\left(t\right)=-R\left(t\right)\Rightarrow f\left(t\right)=-\frac{dR\left(t\right)}{dt}$$ (16)

$$\int_{0}^{t_{0}}\text{t}\times \text{f}\left(\text{t}\right)\text{dt}=\int_{0}^{t_{0}}\text{t}\times \left(-\frac{dR\left(t\right)}{dt}\right)\text{dt}=\int_{0}^{t_{0}}-\text{tdR}\left(\text{t}\right)$$ (17)

By integrating Eq (17) using the partial method, we will have:

$$\{\begin{array}{c} u=-t\\ du=dR\left(t\right) \end{array}\Rightarrow \{\begin{array}{c} du=-dt\\ u=R\left(t\right) \end{array}$$ (18)

$$\int_{0}^{t_{0}}-\text{tdR}\left(\text{t}\right)=-tR{\left(t\right)}_{0}^{t_{0}}+\int_{0}^{t_{0}}\text{R}\left(\text{t}\right)\text{dt}$$ (19)

$$\int_{0}^{t_{0}}\text{t}\times \text{f}\left(\text{t}\right)\text{dt}=-t_{0}\text{R}\left(t_{0}\right)+\int_{0}^{t_{0}}\text{R}\left(\text{t}\right)\text{dt}$$ (20)

Based on Eqs (15) and (20), we will have:

$$MTTF\left(t_{0}\right)=\frac{1}{R\left(t_{0}\right)}\left(MTTF-\left(-t_{0}\text{R}\left(t_{0}\right)+\int_{0}^{t_{0}}\text{R}\left(\text{t}\right)\text{dt}\right)\right)-t_{0}$$ (21)

$$MTTF\left(t_{0}\right)=\frac{MTTF-\int_{0}^{t_{0}}R\left(t\right)}{R\left(t_{0}\right)}$$ (22)

Since we have the following for MTTF:

$$MTTF=\int_{0}^{\infty }\text{tf}\left(\text{t}\right)\text{dt}=\int_{0}^{\infty }R\left(t\right)dt$$ (23)

Then, based on Eqs (22) and (23), the general formula of the (RUL (t)) can be expressed as Eq (24).

$$MTTF\left(t_{0}\right)=\frac{MTTF-\int_{0}^{t_{0}}R\left(t\right)}{R\left(t_{0}\right)}=\frac{\int_{0}^{\infty }R\left(t\right)dt-\int_{0}^{t_{0}}R\left(t\right)dt}{R\left(t_{0}\right)}=\frac{\int_{t_{0}}^{\infty }R\left(t\right)dt}{R\left(t_{0}\right)}$$ (24)

### Case of study

The Sungun copper mine is located 70 km northwest of Ahar and 105 km northeast of Tabriz, East Azerbaijan Province, Iran. By reviewing all loading systems in the Sungun copper mine, a Komatsu PC-1250 excavator was selected as the case of study. The limitations and hypotheses of the research can be stated as follows:

1. The operational phases were only studied and, if needed, the maintenance phase was also included but not the design phase.
2. The study was focused on the environment of mine system, and other environments out of the mine system (e.g., climate, transportation) were not studied.
3. Previously recorded data were used to predict system performance indicators.
4. A significant priority in choosing a load system was its operational status and the possibility of RUL analysis for it.

**Fig 3:**
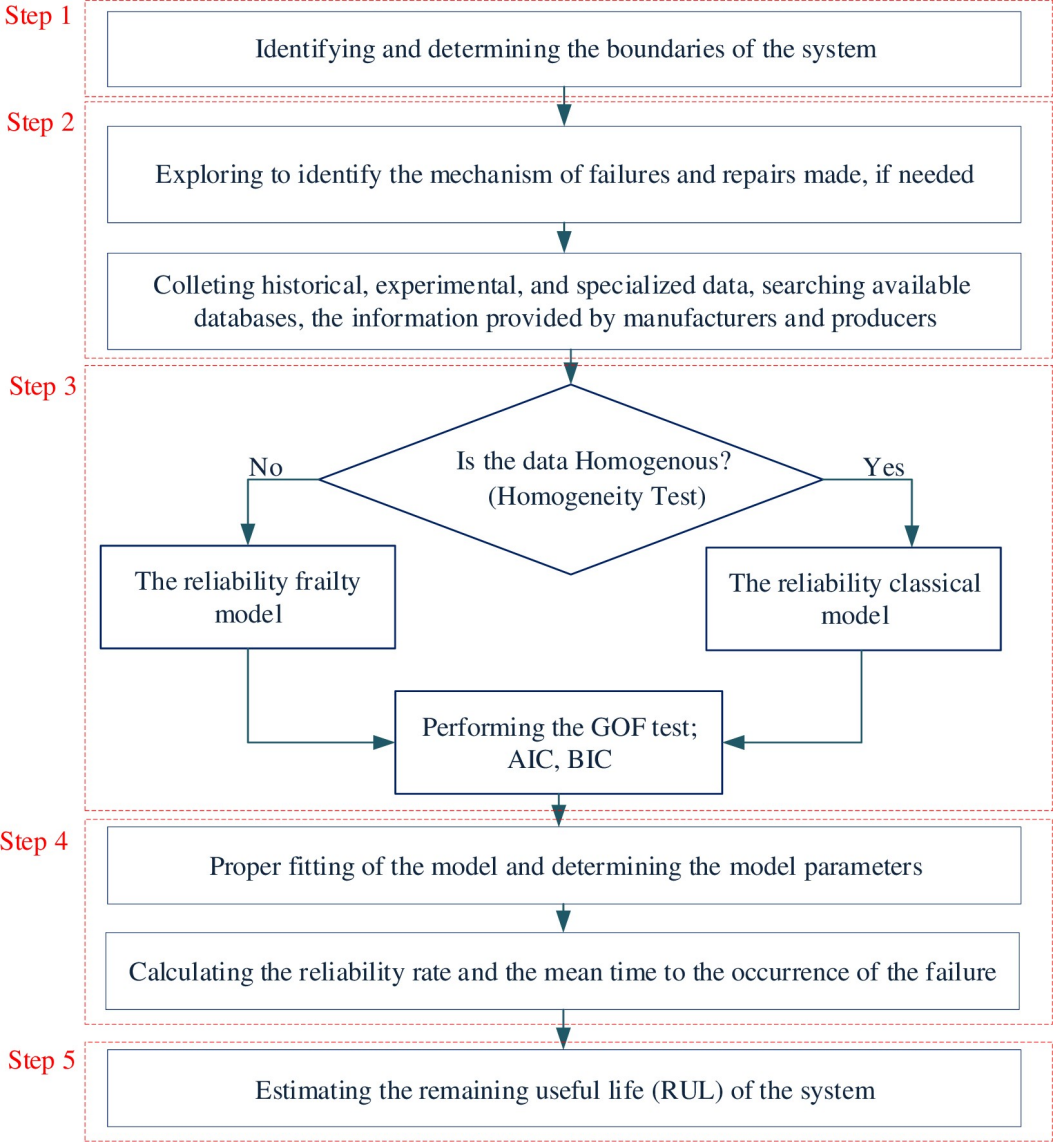
A conceptual framework of the RUL based on the reliability analysis in the frailty model.

Fig 3 shows a conceptual framework of the research in five steps.

Step 1: Determining the boundaries of the mine system.
Step 2: Identifying the mechanism of failures and gathering the required data.
Step 3: Assessing the homogeneity hypothesis to select the appropriate model and performing the goodness of fit (GOF) test to select the proper function for each model, including:
  - Homogeneity test
  - The reliability analysis
  - The reliability-based frailty model
  - The GOF, AIC (Akaike information criterion), and BIC (Bayesian information criterion) tests.
Step 4: Determining the model parameters and calculating the reliability rate.
Step 5: Estimating the RUL of the mine system

## Results

### The excavator database

Since the first step of the proposed algorithm has been performed in the field, this step was rendered, and the second step was described in relation to the excavator database. As can be seen in Eq (24), the RUL value was defined based on the duration of the operational system and its reliability. Thus, the required time data would be the type of time between failures (TBF). Such data were gathered from various sources such as recorded documents (notes of the maintenance group, mechanics group, daily reports, etc.), archived documents (previous reports, machinery manuals, etc.), appointments and interviews, and direct observations (see Supporting Information (S1 Data)). The data of the excavator were recorded three times a day in sheets containing information such as the reports row, device code, operational position, operational level (level of stairs from the sea level), the number of services (depending on the type of load-bearing dump truck: 100 tons, 32 tons), type of rock, working hours, and the number of stops (under repair, not in use but ready to use). The excavator had been working from the beginning of February 2018 to the end of September 2018. Therefore, the data were collected in eight months and sorted consecutively. For more information see Supporting Information (S2 Data).

### The heterogeneity test

Likelihood ratio (LR), AIC, and BIC tests were proposed to test the heterogeneity and to examine the opposite hypothesis (H1) suggesting the presence of heterogeneity versus the zero hypotheses (H0) indicating the absence of heterogeneity (*θ* = 0). Results from the AIC and test present an excellent performance for small values of heterogeneity, while the BIC test shows a proper performance for larger values [21, 32, 33]. We used the LR test in this study, which evaluates the absence of heterogeneity. If the basic hazard rate function is the Weibull model, the maximum LR is as follows:

$$R=2\left(\ln L\left(\hat{\lambda },\;\hat{\beta },\hat{\eta },\;\hat{\theta }\right)-lnL\left({\hat{\lambda }}_{0},{\hat{\beta }}_{0},{\hat{\eta }}_{0},\;0\right)\right)$$ (25)

From Eq (25), $\hat{\lambda }$ and $\hat{\beta }$are the parameters of the basic function. $\hat{\eta }$ and $\hat{\theta }$ are the regression coefficient for observed risk factors and the degree of heterogeneity, respectively. These parameters were estimated by maximizing the complete likelihood function. Since $\hat{\eta }=0$ is not within the parameter space ($\hat{\eta }<0$ is not allowed), thus, R does not have a normal convex chi-square ($\chi_{1}^{2}$) distribution. Indeed, $\hat{\eta }$ has asymptotically chi-square distribution with a degree of freedom of 1 ($\chi_{1}^{2}$) and the probability mass of 0.5 at R = 0. This result implies that at the confidence level of 5%, (H0), the absence of heterogeneity will be rejected if *R* ≥ 2.706. Moreover, under the minimal repairs strategy, we can use the PLP model as the intensity function. Assuming the PLP model, as an intensity function of the failure to examine the presence of the significant value of heterogeneity in the units (subsystems), we can use the following steps in the LR test [21]:

1. In the first step, the H0 equals to *H*_0_: *λ*_1_ = *λ*_2_, *λ*_*m*_ = *λ*_0_, *β*_1_ = *β*_2_, *β*_*m*_ = *β*_0_, which was tested versus the H1(*H*_1_: *λ*_1_ ≠ *λ*_2_, *β*_*m*_ ≠ *β*_0_, *β*_1_ ≠ *β*_2_, and *λ*_*m*_ ≠ *λ*_0_).
2. In the second and third steps, normal *λ* and non-normal *β* and non-normal *λ* and normal *β* were obtained, respectively [34].

From the excavator data, the value of R is 3.96, calculated by the STATA software (Eq 26).

$$R=2\left(\ln\;L\left(\overset{\wedge }{\lambda },\;\overset{\wedge }{\beta },\;\overset{\wedge }{\eta },\;\overset{\wedge }{\theta }\right)-\ln\text{L}\left({\overset{\wedge }{\lambda }}_{0},\;{\overset{\wedge }{\beta }}_{0},\;{\overset{\wedge }{\eta }}_{0},\;0\right)\right)=3.96$$ (26)

For this value of R from the test, the p-value is 0.023, indicating the effect of “unobserved heterogeneity” on the excavator reliability. It can be concluded that reliability analysis in the frailty model tends to the left side of the proposed algorithm in Fig 3.

### Model fitting

BIC and AIC criteria were used as the statistics of the GOF test. These two criteria were relied on the compare data from the maximum LR to select the appropriate model. These two criteria were formulated as follows:

AIC=−2×ln(likelihood)+2×k (27)

BIC=−2×ln(likelihood)+ln(N)×k (28)

From Eqs (27) and (28), k represents the number of parameters estimated, and N indicates the number of observations (failures). The results of both criteria were analyzed together since BIC is more conservative than AIC. Both criteria were recognized as estimators that are capable of fitting the model by calculating the negative value (−2 × *ln*(*likehood*)), and positive values (2 × *k*) for AIC and (*ln*(*N*) × *k*) for BIC. The model with the smallest values of AIC and BIC criteria is the most appropriate model fitting [35–38].

Table 1 presents the values of AIC and BIC for classic and frailty models, and three functions of Weibull, exponential, and Gompertz. In the meantime, the exponential function with 169.43 and 371.53 values for AIC and BIC, respectively, has the lowest value for the classic model. It seems to be the best function to describe the failure behavior. In the case of the frailty model, the lowest AIC value belongs to the Weibull function (i.e., 169.48). For the BIC (i.e., 175.7612), the Weibull-Frailty function was selected as the appropriate model. It is due to the values of Weibull, and exponential functions are close to each other, and the AIC has the lowest value among other functions

**Table 1:** The GOF values of classical and frailty models for the excavator failure data.

Model	Functions	AIC	BIC
**Classic**	Weibull	171.43	175.622
	Exponential	169.44	171.533
	Gompertz	171.13	175.321
**Frailty**	Weibull-frailty	169.48	175.7612
	Exponential-frailty	171.03	175.2224
	Gompertz-frailty	171.43	175.622

Table 2 shows the parameter of the classical-exponential model as a constant value with a mean coefficient of -4.601. From Table 2, the third column indicates the standard error value of the calculated coefficient. The fourth column is the value of the z coefficient by considering H0. As a result, the effect of z coefficient on the function equals to zero, as the corresponding hazard rate equals to one. It can be seen in the fifth column of Table 2 that the p-value of the z-test is zero at a confidence level of 0.05 in the fifth column. Given the zero value, in this case, the corresponding H0 will be rejected. The last two columns also contain the values of the calculated coefficient at the confidence level of 95% for subsequent analyses.

**Table 2:** The exponential function in the classical model for the excavator’s failure data.

Parameter	Coef.	Std. err.	z	P>|z|	[95% Conf. interval]	
Cons.	-4.061	0.143	-	0.000	-4.881	-4.321

Table 3 shows the value of the λ parameter in the classical-exponential model for the excavator’s failure data. The λ is calculated from Eq (29) and obtained data from Table 2.

$$\lambda =\frac{1}{\text{exp}\left(-cons\right)}=0.010039851$$ (29)

**Table 3:** The parameter of λ in the classical-exponential model for the excavator’s failure data.

Parameter	Coef.	Std. err.	z	P>|z|	[95% Conf. interval]	
λ	0.010	0.001	7.000	0.000	0.007	0.013

Also, Table 4 presents the parameters of the classical Weibull-frailty model for the excavator’s failure data. From Table 4 and Eq (7), θ or the variance of the gamma function equals to 3.575 for the reliability analysis in frailty model. Therefore, the only remaining part of the reliability analysis is to determine the Weibull function.

**Table 4:** The parameters of the classical Weibull-frailty function for the excavator failure data.

Parameter	Coef.	Std. err.	z	P>|z|	[95% Conf. Interval]	
Cons	-9.634667	4.369024	-2.21	0.027	-18.1978	-1.071537
/ln_p	1.036	0.574	1.8	0.071	-0.089	2.161
/ln_the	1.274	0.853	1.49	0.135	-0.399	2.947
P	2.817	1.617			0.914	8.679
1/p	0.355	0.204			0.115	1.094
Theta	3.575	3.051			0.671	19.043

According to the p-shape parameter and the constant value (cons.), Eq (30) provides the results of Weibull function, as one-parameter Weibull.

$$R=exp\left(-t^{p}\right)=exp\left(-t^{2.817}\right)$$ (30)

Eqs (31) and (32) were applied to achieve the parameters of α and β. To unify and convert these parameters in a general form of the Weibull function (Table 5), results from Table 4 were used to achieve the values of 2.817 and 30.565 for the parameters of α and β, respectively.

α=p=2.817 (31)

$$\beta =exp\left(-\frac{cons}{\alpha }\right)=30.565$$ (32)

**Table 5:** The parameters of the Weibull-frailty function for the excavator’s failure data.

Parameter	Coef.	Std. err.	z	P>|z|	[95% Conf. Interval]	
α	2.817	1.617	1.740	0.082	-0.353	5.987
β	30.565	13.562	2.250	0.024	3.983	57.147

### The reliability analysis of the excavator

The reliability analysis in the classic-exponential and the Weibull-frailty models is shown as Eqs (33) and (34), respectively.

R(t)=exp(−0.010039851×t) (33)

$$R_{\theta }\left(t\right)={\left[1-\theta ln\left\{e^{-{\left(\frac{t}{\beta }\right)}^{\alpha }}\right\}\right]}^{\frac{-1}{\theta }}$$ (34)

After substituting the relevant values, we will have:

$$R_{\theta }\left(t\right)={\left[1-3.575ln\left\{e^{-{\left(\frac{t}{30.565}\right)}^{2.817}}\right\}\right]}^{\frac{-1}{3.575}}$$ (35)

**Fig 4:**
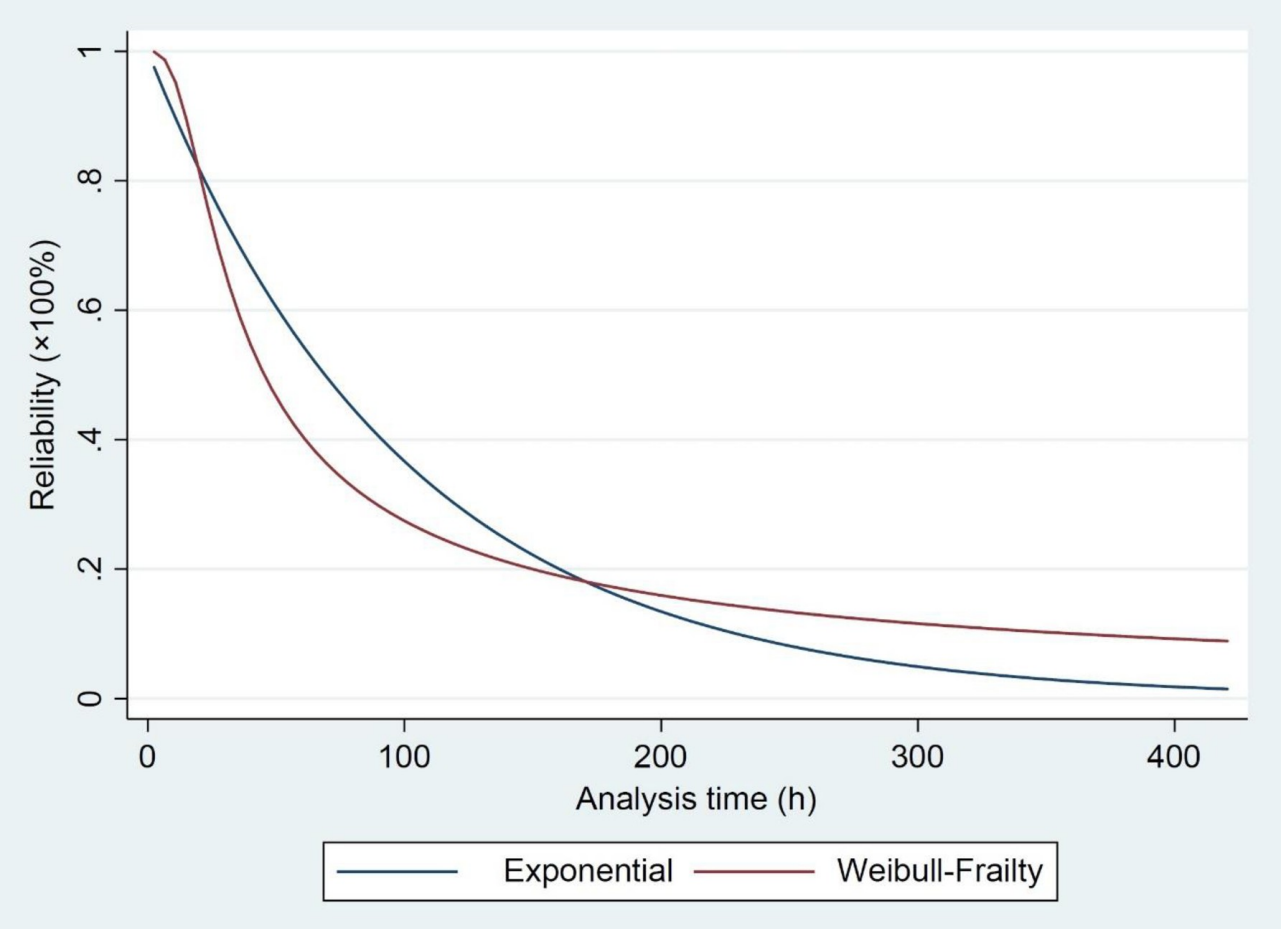
The reliability analysis in the classic-exponential model compared to the Weibull-frailty-model.

From Eq (35), Fig 4 shows the reliability analysis for two models of the classic-exponential and the Weibull-frailty. As shown in Fig 4, the failure value changes the reliability behavior. In the reliability of less than 80%, the mine system with the failure rate shows a better performance compared to the state without the failure rate. When the reliability is between 80% and 20%, the system performance decreases. The reliability of less than 20%, the mine system performs better than the range of 20–80%. These changes indicate the effects of applying or ignoring the impact of the failure rate in the reliability analysis and the behavior of the mine system.

### The RUL of the excavator

As mentioned in sub-section 2.3, Eq (24) was used to estimate the RUL of the excavator. The results from subsections 3.2 and 3.3 showed that the reliability analysis in the classical model is an exponential function. Thus, the RUL of the excavator would be as Eq (36).

$$RUL\left(t_{0}\right)=\frac{\int_{t_{0}}^{\infty }\text{exp}\left(-0.010039851\times t_{0}\right)dt}{\text{exp}\left(-0.010039851\times t_{0}\right)}$$ (36)

**Fig 5:**
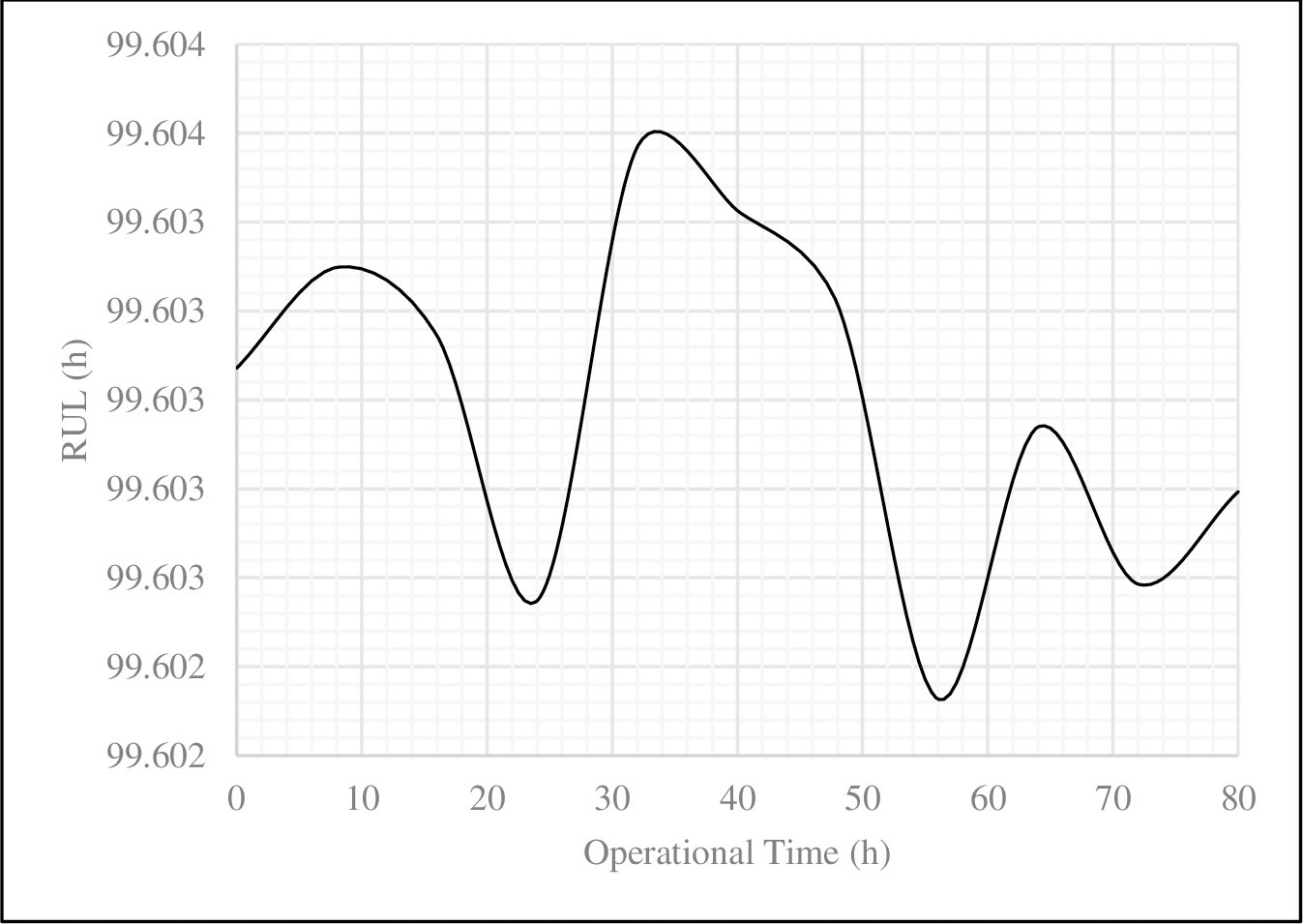
The plot of RUL values during 80 operational hours of the excavator in the classical-exponential model.

Table 6 and Fig 5 show the calculated values of the RUL during 80 operational hours of the excavator.

**Table 6:** RUL values during 80 operational hours of the excavator in the classical-exponential model.

Performance index	*t*_*0*_ *(h)*										
	0	8	16	24	32	40	48	56	64	72	80
**Reliability**	1.000	0.923	0.852	0.786	0.725	0.669	0.618	0.570	0.526	0.485	0.448
**RUL (h)**	99.603	99.603	99.603	99.603	99.604	99.603	99.603	99.602	99.603	99.603	99.603

**Fig 6:**
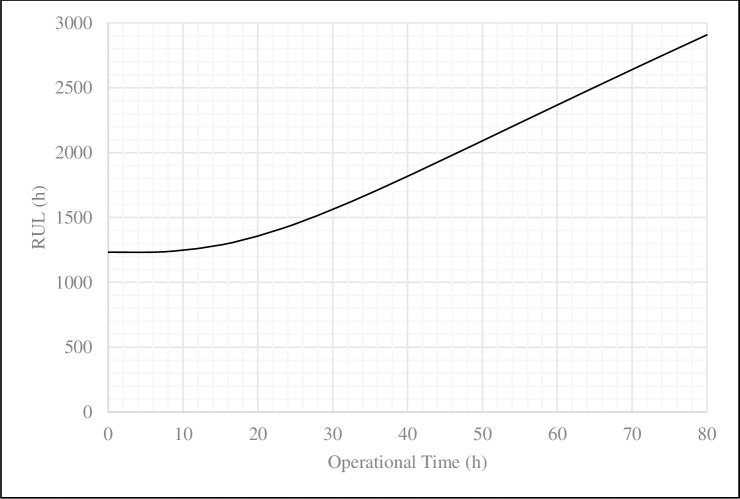
The plot of RUL values during 80 operational hours of the excavator in the Weibull-frailty model.

From Eq (24) in subsection 2.3, the RUL values depend on the reliability analysis and also refer to Eq (35) for the Weibull-frailty model. Therefore, the RUL values of the excavator in the Weibull-frailty model would be as Eq (37). Table 7 and Fig 6 show the calculated values of the RUL during 80 operational hours of the excavator in the Weibull-frailty model. The reliability analysis using the Weibull-frailty model revealed that the mine system would be improved in the long term, and thereby, it does not require any maintenance for the time being. In contrast, the classic-exponential model presents a chaotic situation of the mine system, and it was not possible to make any decision accordingly.

RUL(t0,α)=∫t0∞[1−3.575ln{e−(t030.565)2.817}]−13.575dt[1−3.575ln{e−(t030.565)2.817}]−13.575 (37)

**Table 7:** RUL values during 80 operational hours of the excavator in the Weibull-frailty model.

Performance index	*t*_*0*_ *(h)*										
	0	8	16	24	32	40	48	56	64	72	80
**Reliability**	1.000	0.99	0.94	0.85	0.75	0.66	0.59	0.53	0.48	0.44	0.41
**RUL (h)**	1232.04	1237.62	1299.24	1429.96	1610.75	1817.79	2035.74	2256.66	2476.66	2693.98	2907.8

### Conclusions

The present study was designed to determine the RUL values of the excavator in the Sungun copper mine. The conceptual framework relied on the reliability analysis in two classical and frailty models. The frailty model was also used to evaluate the impact of unobserved environmental conditions on the RUL values. The presence of heterogeneity was detected in the frailty model by examining the data behavior through a statistical test. The data behavior was evaluated from February to September 2018. Both applied models were fitted to the obtained data from 80 operational hours of the Komatsu PC-1250 excavator. Plotting the results from the reliability analysis of two models demonstrated that the mine system with the frailty model performs better than the classical model before reaching the reliability of 80%. When the reliability was ranged between 80% and 20%, the system performance decreased. The mining system showed a better performance in the reliability of less than 20%. These changes indicate the impact of the failure rate in the reliability analysis and the behavior of the mine system. As a result of plotting the RUL values, the frailty model shows a coherent with the operational time of the excavator, caused the improvement of the mine system, while the classical model demonstrates a sinusoid variation.

## Supporting information

S1 Data Collected data in relation to the type of time between failures (TBF). (XLSX)

S2 Data Weather conditions recorded for working hours of the Komatsu excavator from the beginning of February 2018 to the end of September 2018. (XLSX)

## References

[1] Congressional Research Service, Informing the legislative debate since 1914, Iran sanctions, RS20871, April 2020. https://crsreports.congress.gov

[2] Li N, Gebraeel N, Lei Y, Bian L, Si X. Remaining useful life prediction of machinery under time-varying operating conditions based on a two-factor state-space model. Reliability Engineering & System Safety 2019, 186, 88–100.

[3] Mahamad AK, Saon S, Hiyama T. Predicting remaining useful life of rotating machinery based artificial neural network. Comput Math Appl 2010, 60 (4), 1078–87.

[4] Tian Z. Lin D. Wu B. Condition-based maintenance optimization considering multiple objectives. J Intell Manuf 2012, 23, 333–340. 10.1007/s10845-009-0358-7

[5] Watson G, Wells W. On the possibility of improving the mean useful life of items by eliminating those with short lives. Technometrics 1961, 3 (2), 281–298.

[6] Elsayed EA. 2003 Mean Residual Life and Optimal Operating Conditions for Industrial Furnace Tubes In Case Studies in Reliability and Maintenance (eds Balding D.J., Bloomfield P., Cressie N.A., Fisher N.I., Johnstone I.M., Kadane J., Ryan L.M., Scott D.W., Smith A.F., Teugels J.L., Blischke W.R. and Murthy D.N.P.). 10.1002/0471393002.ch22

[7] Xie M, Goh TN, Tang Y. On changing points of mean residual life and failure rate function for some generalized Weibull distributions. Reliability Engineering & System Safety 2004, 84 (3), 293–299.

[8] Xie M, Tang Y, Goh TN. A modified Weibull extension with bathtub shaped failure rate function. Reliability Engineering & System Safety 2002, 73, 279–285.

[9] Liu L, Wang S, Liu D, Peng Y. Quantitative selection of sensor data based on improved permutation entropy for system remaining useful life prediction. Microelectronics Reliability 2017, 75, 264–270.

[10] Ghodrati B, Kumar U, Ahmadzadeh F. Remaining useful life estimation of mining equipment- A case study. The International Symposium on Mine Planning and Equipment Selection 2012. Proceedings of MPES 2012, New Delhi, India. http://urn.kb.se/resolve?urn=urn%3Anbn%3Ase%3Altu%3Adiva-32391

[11] Schuh P, Stern H, Tracht K. Integration of expert judgment into remaining useful lifetime prediction of components. Procedia CIRP 2014, 22, 109–114.

[12] Misra KB. Reliability Engineering: A Perspective In Handbook of Performability Engineering, Springer, 2008, pp. 253–289.

[13] IEC 60050—International Electrotechnical Vocabulary, details for IEV number 191-04-01: ‘failure’. http://www.electropedia.org/iev/iev.nsf/display?openform&ievref=191-04-01 [Accessed: 24-Jan-2019].

[14] Diallo C, Ait-Kadi D, Chelbi A. Integrated Spare Parts Management in Handbook of Maintenance Management and Engineering, Ben-Daya M., Duffuaa S. O., Raouf A., Knezevic J., and Ait-Kadi D., Eds. Springer London, 2009, pp. 191–222.

[15] Dhillon BS. Mining equipment reliability, maintainability, and safety. Springer, 2008.

[16] Pham H. System software reliability. Springer, 2006.

[17] Barlow RE, Proschan F. Mathematical theory of reliability 1996, 17, Siam.

[18] IEC 60050—International Electrotechnical Vocabulary—Details for IEV number 191-02-06: ‘reliability (performance).http://www.electropedia.org/iev/iev.nsf/display?openform&ievref=191-02-06. (Accessed on 24-Jan-2019).

[19] Pijnenburg M. Additive hazards models in repairable systems reliability. Reliability Engineering & System Safety 1991, 31(3), 369–390.

[20] Dhillon BS. Bibliography of literature on mining equipment reliability. Microelectronics Reliability 1986, 26 (6), 1131–1138.

[21] Garmabaki HS, Ahmadi A, Block J, Pham H, Kumar U. A reliability decision framework for multiple repairable units. Reliability Engineering & System Safety 2016, 150, 78–88.

[22] Garmabaki HS, Ahmadi A, Mahmood YA, Barabadi A. Reliability Modelling of Multiple Repairable Units. Quality and Reliability Engineering International 2016, 32 (7), 2329–2343.

[23] Asfaw ZG, Lindqvist BH. Unobserved heterogeneity in the non-homogeneous Poisson process. Reliability Engineering & System Safety 2015, 134, 59–65.

[24] Slimacek V, Lindqvist BH. Nonhomogeneous Poisson process with nonparametric frailty and covariates. Reliability Engineering & System Safety 2017, 167, 75–83.

[25] Cha J H, Finkelstein M. “Some notes on unobserved parameters (frailties) in reliability modeling,” Reliability Engineering & System Safety 2014, 123, 99–103.

[26] Gutierrez RG. “Parametric frailty and shared frailty survival models,” Stata Journal 2002, 2 (1), 22–44.

[27] Xiongzi C, Jinsong Y, Diyin T, Yingxun W. Remaining useful life prognostic estimation for aircraft subsystems or components: A review. presented at the Electronic Measurement & Instruments (ICEMI), 10th International Conference, 2011, 2, 94–98.

[28] Lee M-LT, Whitmore GA. “Threshold regression for survival analysis: modeling event times by a stochastic process reaching a boundary. Statistical Science 2006, 501–513.

[29] Wang W. A prognosis model for wear prediction based on oil-based monitoring. Journal of the Operational Research Society 2007, 58 (7), 887–893.

[30] Wang W., Zhang W. An asset residual life prediction model based on expert judgments. European Journal of Operational Research 2008,188 (2), 496–505.

[31] Mazhar M., Kara S., Kaebernick H. Remaining life estimation of used components in consumer products: Life cycle data analysis by Weibull and artificial neural networks. Journal of Operational Research 2007, 25 (6), 1184–1193.

[32] Lawless J. F. Regression methods for Poisson process data. Journal of the American Statistical Association 1987, 82 (399), 808–815.

[33] Brewer M. J., Butler A., Cooksley S. L. The relative performance of AIC, AICC and BIC in the presence of unobserved heterogeneity. Methods in Ecology and Evolution 2016, 7 (6), 679–692.

[34] Giorgio M, Guida M, Pulcini G. Repairable system analysis in presence of covariates and random effects. Reliability Engineering & System Safety 2014, 131, 271–281.

[35] Akaike H. A New Look at the Statistical Model Identification,” in Selected Papers of Akaike H, Parzen E, Tanabe K, and Kitagawa G. Eds. New York, NY: Springer New York, 1998, pp. 215–222.

[36] Raftery E. Bayesian model selection in social research. Sociological methodology 1995, 25, 111–164.

[37] Sakamoto Y, Ishiguro M, Kitagawa G. Akaike information criterion statistics. Dordrecht, The Netherlands: D. Reidel 1986, 83, 902–926.

[38] Schwarz G. Estimating the dimension of a model. The Annals of Statistics 1978, 6 (2), 461–464.

