# Correction: Prediction of remaining useful life (RUL) of Komatsu excavator under reliability analysis in the Weibull-frailty model

**DOI:** 10.1371/journal.pone.0254743

**Published:** 2021-09-10

**Authors:** Awat Ghomghaleh, Reza Khaloukakaie, Mohammad Ataei, Abbas Barabadi, Ali Nouri Qarahasanlou, Omeid Rahmani, Amin Beiranvand Pour

The second corresponding author is not indicated. The correct second corresponding author is Awat Ghomghaleh. Awat Ghomghaleh’s email address is: ghomghale@gmail.com.
